# Integrated clustering signature of genomic heterogeneity, stemness and tumor microenvironment predicts glioma prognosis and immunotherapy response

**DOI:** 10.18632/aging.205018

**Published:** 2023-09-11

**Authors:** Yangyang Wu, Meng Mao, Lin-Jian Wang

**Affiliations:** 1 Advanced Medical Research Center of Zhengzhou University, Zhengzhou Central Hospital Affiliated to Zhengzhou University, Zhengzhou 450007, China; 2 Department of Anesthesiology and Perioperative Medicine, Zhengzhou Central Hospital Affiliated to Zhengzhou University, Zhengzhou 450007, China; 3 Research of Trauma Center, Zhengzhou Central Hospital Affiliated to Zhengzhou University, Zhengzhou 450007, China; 4 Department of Neurosurgery, Zhengzhou Central Hospital Affiliated to Zhengzhou University, Zhengzhou 450007, China

**Keywords:** glioma, genomic heterogeneity, stemness, tumor microenvironment, risk signature

## Abstract

Background: Glioma is the most frequent primary tumor of the central nervous system. The high heterogeneity of glioma tumors enables them to adapt to challenging environments, leading to resistance to treatment. Therefore, to detect the driving factors and improve the prognosis of glioma, it is essential to have a comprehensive understanding of the genomic heterogeneity, stemness, and immune microenvironment of glioma.

Methods: We classified gliomas into various subtypes based on stemness, genomic heterogeneity, and immune microenvironment consensus clustering analysis. We identified risk hub genes linked to heterogeneous characteristics using WGCNA, LASSO, and multivariate Cox regression analysis and utilized them to create an effective risk model.

Results: We thoroughly investigated the genomic heterogeneity, stemness, and immune microenvironment of glioma and identified the risk hub genes RAB42, SH2D4A, and GDF15 based on the TCGA dataset. We developed a risk model utilizing these genes that can reliably predict the prognosis of glioma patients. The risk signature showed a positive correlation with T cell exhaustion and increased infiltration of immunosuppressive cells, and a negative correlation with the response to immunotherapy. Moreover, we discovered that SH2D4A, one of the risk hub genes, could stimulate the migration and proliferation of glioma cells.

Conclusions: This study identified risk hub genes and established a risk model by analyzing the genomic heterogeneity, stemness, and immune microenvironment of glioma. Our findings will facilitate the diagnosis and prediction of glioma prognosis and may lead to potential treatment strategies for glioma.

## INTRODUCTION

Central nervous system (CNS) malignancies are among the cancers with the poorest prognosis [1]. Glioma is the most common primary central nervous tumor in the brain, accounting for approximately 81% of malignant brain tumors. Gliomas usually originate from glial cells or precursor cells and progress to astrocytomas, oligodendrogliomas, ependymomas, or oligoastrocytomas [2, 3]. According to the previous classification scheme established by the World Health Organization, primary gliomas are classified as grades I to IV [4]. This grading system reflects the malignancy of the tumor, and in general, a higher grade is associated with a worse prognosis. WHO grade I indicates that slow-growing lesions are usually associated with a good prognosis, while WHO grade IV is recognized as highly malignant [5]. WHO grade II and III gliomas, including astrocytomas, oligodendrogliomas, and mixed oligodendrogliomas are defined as low-grade gliomas (LGG). Generally, low-grade gliomas (LGG) show some sensitivity to treatment and have a better prognosis [6, 7]. World Health Organization grade IV gliomas (glioblastomas, GBM) are highly malignant and also the most common gliomas, accounting for approximately 45% of all gliomas [8, 9]. At present, molecular pathological features (such as mutation status of isocitrate dehydrogenase gene IDH1 or IDH2) are used to classify adult glioma. IDH-mutated gliomas usually present with a lower histological grade and have a better prognosis. Median survival is approximately 12 years [10]. In contrast, IDH wild-type gliomas usually present as glioblastomas (GBM) with a poor prognosis and a median survival of only 12 - 15 months [11–13].

Traditional cancer treatments, such as surgery, chemotherapy, and radiation therapy, have shown limited improvement in the prognosis of patients with glioma. The main reason for the limited therapeutic progress in glioma is the blood-brain barrier consisting of endothelial cells, capillaries, and basement membrane, which prevents most anti-tumor drugs from entering the brain. Thus, although many cancer therapies have been developed, few drugs have been approved for the treatment of glioma [14, 15]. Immunotherapy is currently at the forefront of cancer treatment. Even though immune checkpoint inhibitors (ICI) can penetrate the blood-brain barrier, they still do not improve the prognosis of glioma [16, 17]. Gliomas are characterized by a high degree of heterogeneity and the ability to proliferate aggressively [11]. The heterogeneity of gliomas allows them to adapt to challenging microenvironments, leading to treatment resistance [18, 19]. For patients with glioblastoma, recurrences and resistance to treatment can result in a short average survival time after treatment. To a large extent, this is due to the molecular heterogeneity of gliomas, which affects the overall prognosis and response to treatment [20, 21]. In addition, the tumor microenvironment is another factor in the development and treatment resistance of glioma. The tumor microenvironment (TME) consists of a variety of non-tumor cells, such as endothelial cells, stromal cells, mesenchymal cells, and immune cells, which play an extremely important role in tumor progression, recurrence, and drug resistance [22–24]. Communication between glioma cells and adjacent cells and the immune environment accelerates the cancer process and contributes to the formation of glioma stem cells, resulting in treatment resistance [21, 25]. Meanwhile, the interaction between tumor microenvironment (TME) and tumor stem cells significantly affects the aggressive proliferation and molecular heterogeneity of tumors [25].

Here, we found that consensus clustering analysis based on tumor stemness, genomic heterogeneity, and immune microenvironment could classify gliomas into distinct subtypes, and the prognosis was significantly different among subtypes. By screening differentially expressed genes, and performing WGCNA, LASSO, and multivariate Cox regression analyses, we identified risk hub genes (RAB42, SH2D4A, and GDF15) associated with glioma stemness, genomic heterogeneity, and the immune microenvironment. The prognostic model constructed using these risk hub genes in this study could predict not only glioma prognosis, but also immunotherapy response in immunotherapy cohort. Although SH2D4A appears to play opposing roles in tumors [26, 27], our experiments demonstrated that knockdown of SH2D4A significantly inhibited the migration and proliferation of glioma cells. In conclusion, we investigated the impact of glioma stemness, genomic heterogeneity, and immune microenvironment-related risk hub genes on glioma and provided new ideas for therapeutic strategies for glioma.

## MATERIALS AND METHODS

### Datasets and samples

The TCGA dataset (Supplementary Table 1) was downloaded from the Xena Browser at the University of California, Santa Cruz (UCSC, https://xenabrowser.net/datapages/) [28]. The clinical data (Supplementary Table 2) and RNA-seq data of the Chinese Glioma Genome Atlas (CGGA) were obtained from the CGGA data portal (http://www.cgga.org.cn/) [29]. The data for the IMvigor210 cohort were loaded from the R package “IMvigor210CoreBiologies” [30].

### Analysis of genomic heterogeneity, stemness, and immune microenvironment

The glioma single nucleotide variant dataset processed by MuTect2 software [31] was downloaded from GDC (https://portal.gdc.cancer.gov/). Tumor mutation burden (TMB) was calculated for each glioma using the tmb function of the R package “maftools”, and microsatellite instability (MSI) for each glioma was obtained from previous studies [32, 33]. Glioma stemness scores based on RNA expression and DNA methylation were calculated according to a previous study [34]. StromalScore, ImmuneScore, and ESTIMATEScore were calculated for each glioma based on gene expression using the R software package “estimate” [35]. The abundance of tumor-infiltrating immune cells in gliomas was analyzed using the CIBERSOR, MCPCOUNTER and EPIC algorithms on the TIMER2 platform (http://timer.cistrome.org/) [36].

### Consensus clustering analysis

Cluster analysis was performed by ConsensusClusterPlus [37], using agglomerative pam clustering with 1-pearson correlation distances, with 10 repetitions for 80% of the samples. The empirical cumulative distribution function plot was used to determine the optimal number of clusters.

### Transfection with siRNA

These siRNAs were synthesized by the Shanghai GenePharma Co. (Supplementary Table 3). U87 MG cells were transfected with negative control siRNA and SH2D4A siRNA according to the manufacturer’s instructions of Lipofectamine 3000 (Invitrogen, L3000015). 48 hours after transfection, U87 MG cells were harvested for subsequent western blot and qRT-PCR experiments to verify their knock-down efficiency.

### Western blot analysis

Western blot analysis was performed according to our previously reported method with minor modifications [38]. In brief, protein extracts were prepared using RIPA lysis buffer (Epizyme) in the presence of 100x EDTA-free Protease Inhibitor Cocktail (Epizyme). Cell lysates were separated through electrophoresis and electro-transferred to polyvinylidene difluoride (PVDF) membranes (Merck Millipore). The membrane was blocked with 5% non-fat milk in TBS solution for 2 h at room temperature and incubated with anti-SH2D4A (15957-1-AP; Proteintech) or anti-α-Tubulin (11224-1-AP; Proteintech) overnight at 4° C. Blots were then washed thrice with TBST and probed for 2 h at room temperature with HRP- conjugated AffiniPure Goat Anti-Rabbit IgG (SA00001-2; Proteintech). Finally, the labeled proteins were detected using the ECL reagent.

### Quantitative real-time PCR

Trizol (Thermo Fisher) was used to extract RNA samples, and then 1 μg of total RNA and NovoScript® Plus All-in-one 1st Strand cDNA Synthesis SuperMix (gDNA Purge) (Novoprotein E047-01B) were used to prepare cDNA according to the manufacturer’s instructions. Thereafter, cDNA was then analyzed by NovoStart® SYBR qPCR SuperMix Plus (Novoprotein E096-01A). Relative gene expression was evaluated using the 2^−ΔΔCT^ method. The GAPDH was utilized as an internal control, and all primer sequences are compiled in Supplementary Table 4.

### Cell proliferation and migration

Cell proliferation was analyzed by Cell Counting Kit-8 (CCK-8) (CellorLab, CX001L) and BeyoClick™ EdU-594 assay kit (Beyotime, C0078S). After 24h of transfection, cell suspensions were planted in 96-well plates. At 24, 48, 72 and 96h, 10ul CCK-8, and 90ul medium were incubated together at 37° C for 2h, then the absorbance at 450 nm was detected by a microplate reader.

For direct observation of the proliferating cells, the 5-Ethynyl-2-deoxyuridine incorporation experiment was also performed according to the specifications. The cells were further incubated with EdU for 2h before fixation, permeabilization, and EdU staining. The cell nuclei were stained with Hoechst33342 for 30min. Finally, the proportion of cells that incorporated EdU was detected using confocal laser microscopy.

To perform migration assays, U87 MG cells were seeded into 6-well plates. Transfection was performed when cell density reached 70-90%. 48 h later the plates were scraped with pipette tips, washed with PBS, and incubated with serum-free medium. The original images and migrated images were obtained using the inversion microscope system. The migrated area was analyzed by Image J software.

### Construction of the risk model

In order to construct a scale-free co-expression network, a weighted correlation network analysis (WGCNA) was performed using the R package “WGCNA”, and four co-expression modules were finally obtained after merging modules with distances less than 0.25. Genes with high connectivity in the clinically important modules were identified as hub genes. LASSO and multivariate regression analyses were then performed sequentially to screen for positive hub genes that were significantly associated with overall survival (OS). Risk scores were calculated as follows:


$$Risk\;score=\sum_{\text{i}=1}^{\text{n}}C{\text{oef}}_{\text{i}}*Exp_{i}$$

### Identification of DEGs and enrichment analysis

We identified differentially expressed genes (DEGs) between different clusters by executing the “limma” package in the R software (p< 0.05 and |FC| ≥ 1.5). Gene enrichment analysis was conducted using Metascape [39].

### Statistical analysis

One-way ANOVA, Wilcox test and t-test were used to analyze the significance of differences in heterogeneity, stemness, gene expression, and infiltration of immune cells in gliomas. LASSO, multivariate Cox regression, and Kaplan-Meier analyses were performed to screen and evaluate the risk signature using the R packages “glmnet” and “survival”. Roc curve was drawn using the R package “survivalROC.” All statistical analyses were performed using GraphPad Prism and R software, and P values less than 0.05 were considered statistically significant.

### Availability of data and material

Bioinformatics datasets presented in this study can be found in online repositories, and the datasets used and/or analyzed during experiments are available from the corresponding author on reasonable request.

### Consent for publication

All authors had final approval of the submitted versions and read the journal’s authorship statement.

## RESULTS

### Consensus clustering analysis of stemness, genomic heterogeneity, and the immune microenvironment in glioma

**Figure 1:**
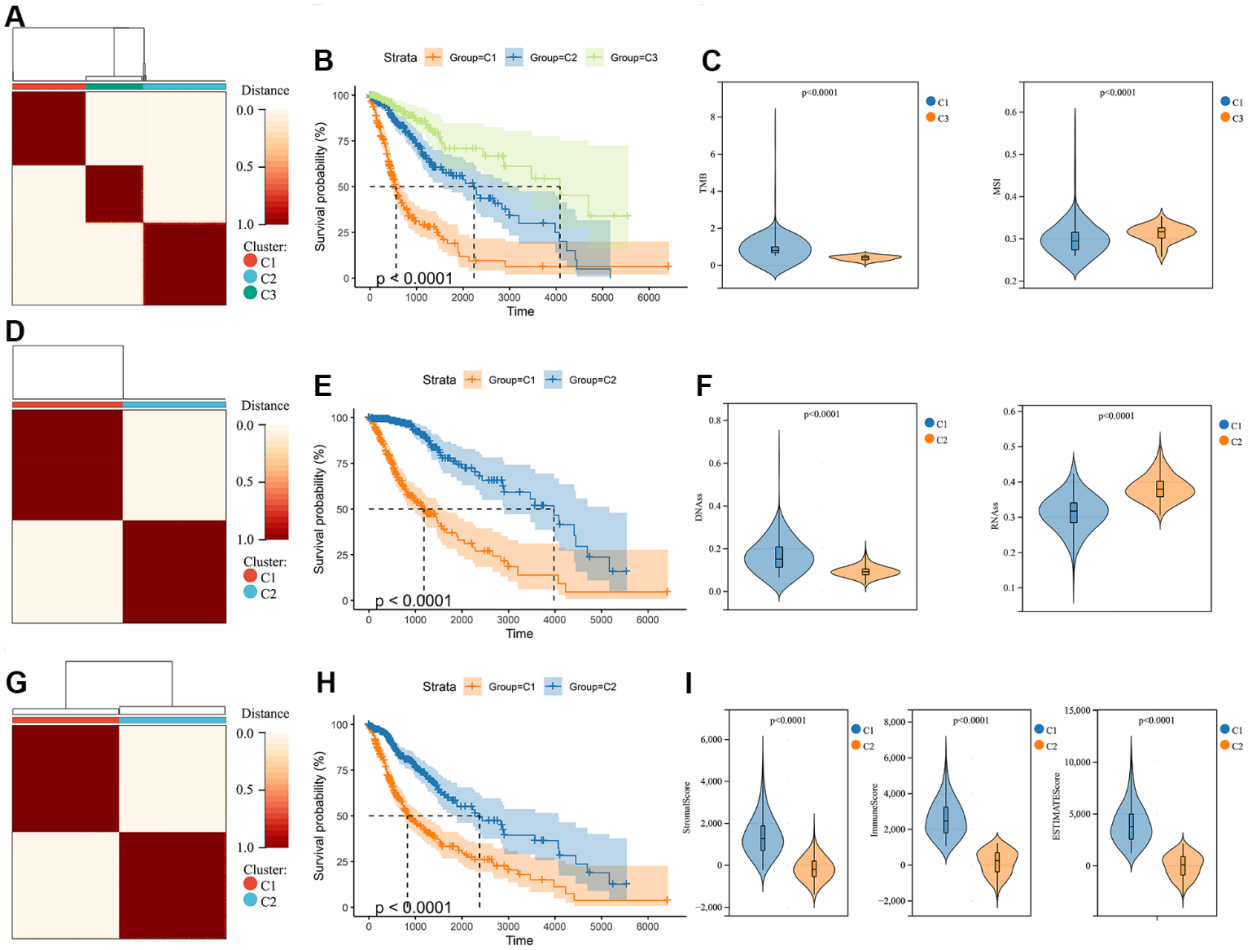
**Consensus clustering analysis in glioma.** (**A**) Consensus clustering was performed based on the genomic heterogeneity of gliomas. (**B**) Kaplan-Meier curves displaying prognostic differences between different clusters. (**C**) The differences in TMB and MSI between clusters. (**D**) Consensus clustering was performed based on the stemness of gliomas. (**E**) Kaplan-Meier curves displaying prognostic differences between different clusters. (**F**) The differences in DNAss and RNAss between clusters. (**G**) Consensus clustering is performed based on the microenvironment of gliomas. (**H**) Kaplan-Meier curves displaying prognostic differences between different clusters. (**I**) The differences in the microenvironment scores between clusters.

The molecular heterogeneity of gliomas significantly affects the overall prognosis of patients and their response to treatment. Three clusters were identified through unsupervised consensus clustering analysis of genomic heterogeneity of gliomas in the TCGA dataset (Figure 1A). We examined the overall survival of the three clusters and found significant differences in prognosis between them. Cluster 1 had the lowest prognosis, while cluster 3 had the highest prognosis (Figure 1B). Significant differences were observed in TMB and MSI between cluster 1, with the lowest prognosis, and cluster 3, with the highest prognosis (Figure 1C). In addition, gliomas were divided into two subtypes based on the unsupervised consensus clustering analysis of stemness scores in the TCGA dataset (Figure 1D). The prognosis of cluster 2 was more favorable than that of cluster 1 (Figure 1E). Assessment of glioma stemness scores based on RNA expression (RNAss) and DNA methylation (DNAss) revealed that cluster 1 had higher DNAss and lower RNAss than cluster 2 (Figure 1F). Tumor microenvironment is a crucial factor in glioma development and treatment resistance. We conducted another cluster analysis to examine the immune microenvironment of glioma. This analysis revealed that the StromalScore, ImmuneScore, and ESTIMATEScore differed significantly between clusters, with cluster 1 having higher scores than cluster 2. Improved prognosis was observed in cluster 2 (Figure 1G–1I).

### Identification of DEGs associated with glioma stemness, genomic heterogeneity, and immune microenvironment

**Figure 2:**
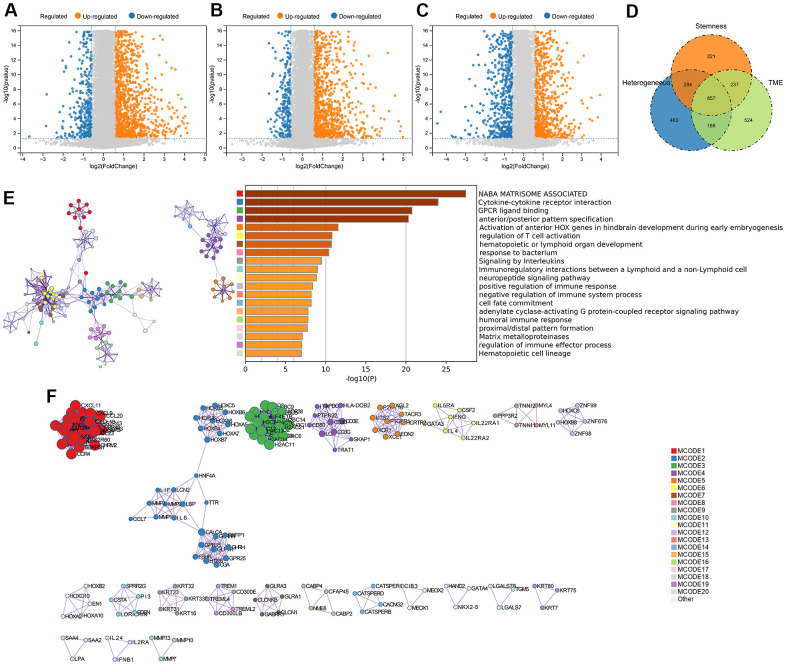
**DEGs screening and enrichment analysis.** (**A**) Differentially expressed genes (Fold change >1.5 and P < 0.05) were screened between different clusters distinguished in genomic heterogeneity, (**B**) stemness, and (**C**) tumor microenvironment, respectively. (**D**) Venn diagram showing the overlap of identified differentially expressed genes. (**E**) Enrichment analysis of the differentially expressed genes. (**F**) Protein-Protein Interaction Networks, PPI.

We quantified gene expression among the clusters divided according to genomic heterogeneity, stemness, and tumor microenvironment, and identified 1792 (412 down-regulated, 1380 up-regulated), 1599 (306 down-regulated, 1293 up-regulated), and 1806 (709 down-regulated, 1097 up-regulated) differentially expressed genes (DEGs), respectively (Figure 2A–2C). And finally, we identified 857 DEGs shared by the three clustering (Figure 2D). Enrichment analysis revealed that differentially expressed genes were involved in immunoregulatory pathways, including Cytokine-cytokine receptor interaction, Signaling by Interleukins, regulation of T cell activation, and Immunoregulatory interactions between a Lymphoid and a non-Lymphoid cell pathway (Figure 2E). We extracted all protein-protein interactions among differentially expressed genes from a PPI data source and constructed a PPI network. MCODE algorithm was applied to this network to identify neighborhoods where proteins are densely connected (Figure 2F). GO enrichment analysis was applied to the network to reveal biological meanings, and the results showed that MCODE 3 was enriched in HDACs deacetylate histones; MCODE 4 was enriched in TCR signaling Translocation of ZAP-70 to Immunological synapse, Phosphorylation of CD3 and TCR zeta chains; MCODE 6 was enriched in JAK-STAT signaling pathway, Signaling by Interleukins, and Cytokine Signaling in Immune system (Figure 2F).

### Screening of risk hub genes

**Figure 3:**
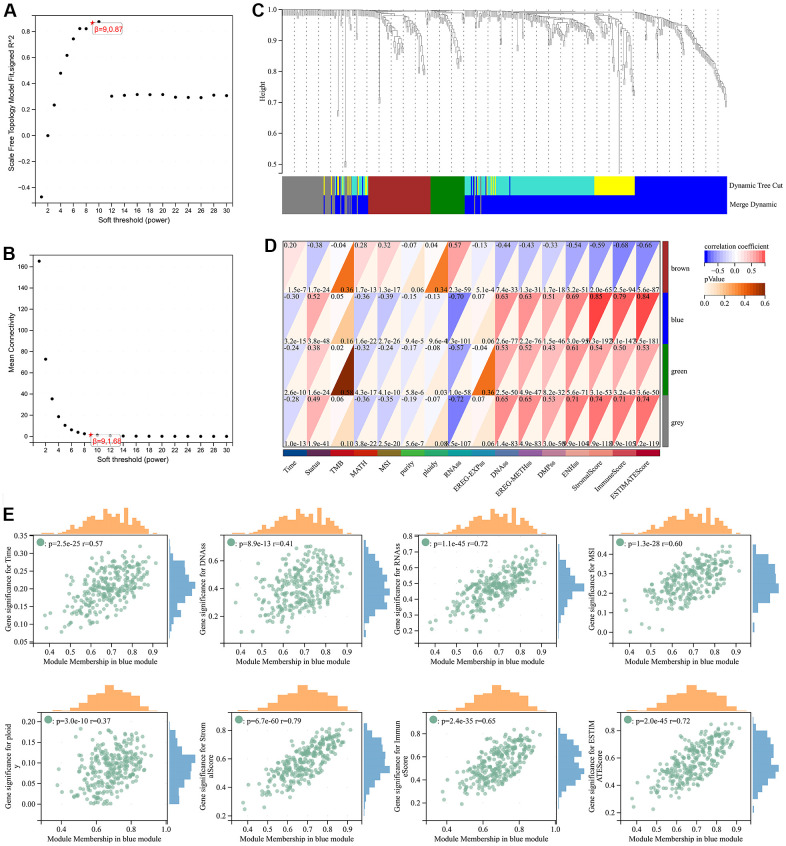
**Weighted correlation network analysis.** (**A**, **B**) Soft-thresholding powers. (**C**) Clustering of module genes in the TCGA cohort. (**D**) Module-trait relationships. (**E**) Scatter plot of correlation between GS and MM. *, P < 0.05; **, P < 0.01; ***, P < 0.001; ****, P < 0.0001.

**Figure 4:**
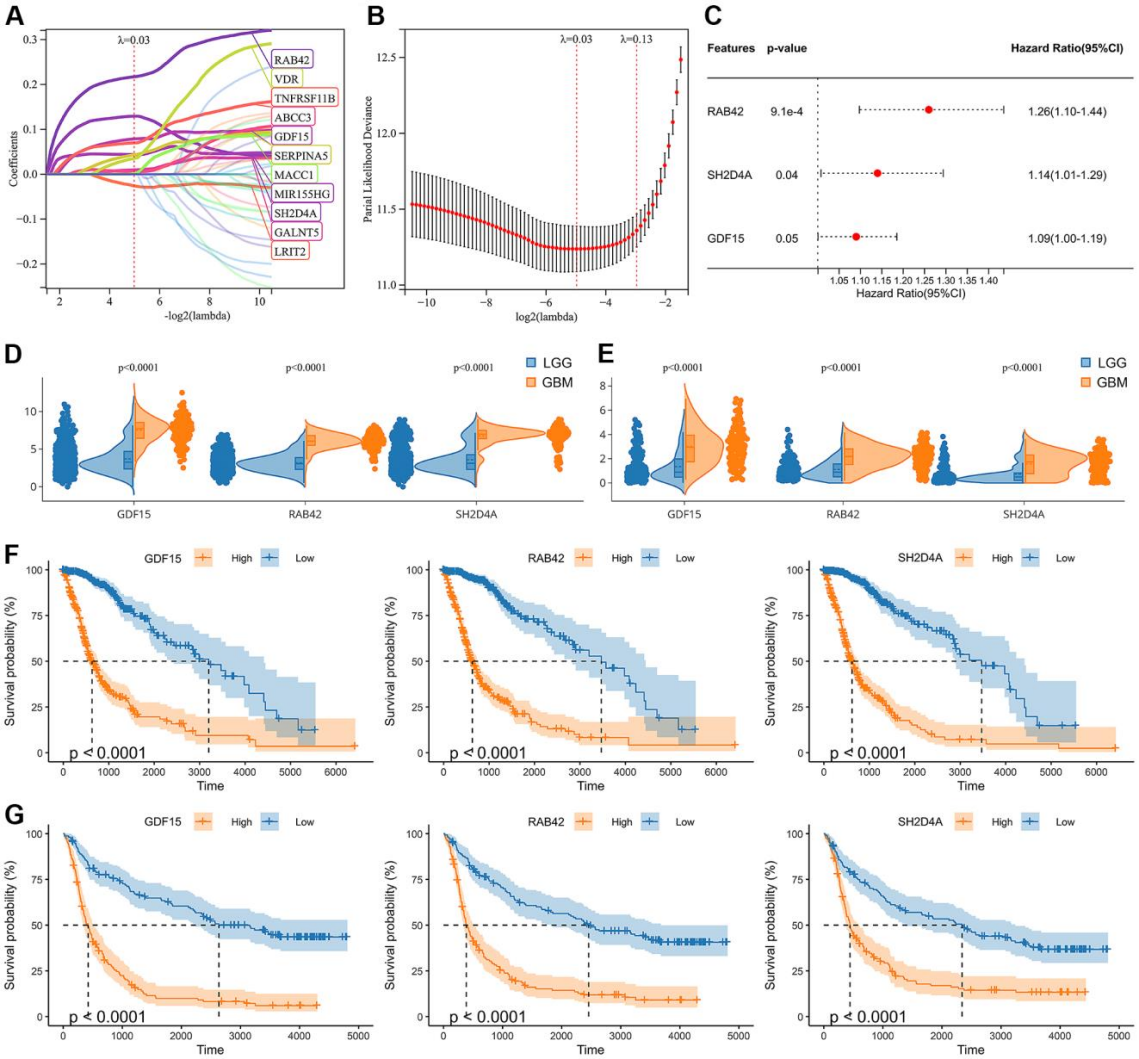
**Identification of risk hub genes related to the overall survival of glioma.** (**A**, **B**) LASSO regression analysis of hub genes in the TCGA cohort. (**C**) Multivariate Cox regression analysis of hub genes in the TCGA cohort. (**D**, **E**) Violin plot showing the expression levels of the 3 risk hub genes between LGG and GBM in the TCGA and CGGA datasets, respectively. (**F**, **G**) Kaplan-Meier curves displayed 3 risk hub genes were significantly related to poor prognosis in the TCGA and CGGA datasets, respectively.

Through WGCNA analysis of integrated data containing DEGs and clinical characteristics (such as genomic heterogeneity, stemness and tumor immune microenvironment), four co-expression modules were finally determined (Figure 3A–3C). We filtered the most relevant module by evaluating its interrelationships with traits and identified the blue module as the most relevant (Figure 3D, 3E). The hub genes of blue module were extracted. Subsequently, LASSO Cox regression analysis and multivariate Cox regression analysis were performed sequentially to filter variables. Ultimately, we selected RAB42, SH2D4A, and GDF15 as risk hub genes (Figure 4A–4C). The expression of three risk hub genes in glioma was investigated in the TCGA and CGGA datasets, and RAB42, SH2D4A, and GDF15 were found to be higher expressed in GBM than in LGG (Figure 4D, 4E). In the TCGA and CGGA datasets, the Kaplan-Meier curve showed that RAB42, SH2D4A, and GDF15 significantly affected the prognosis of glioma, with a poor prognosis at high expression (Figure 4F, 4G).

### Construction of the risk signature

**Figure 5:**
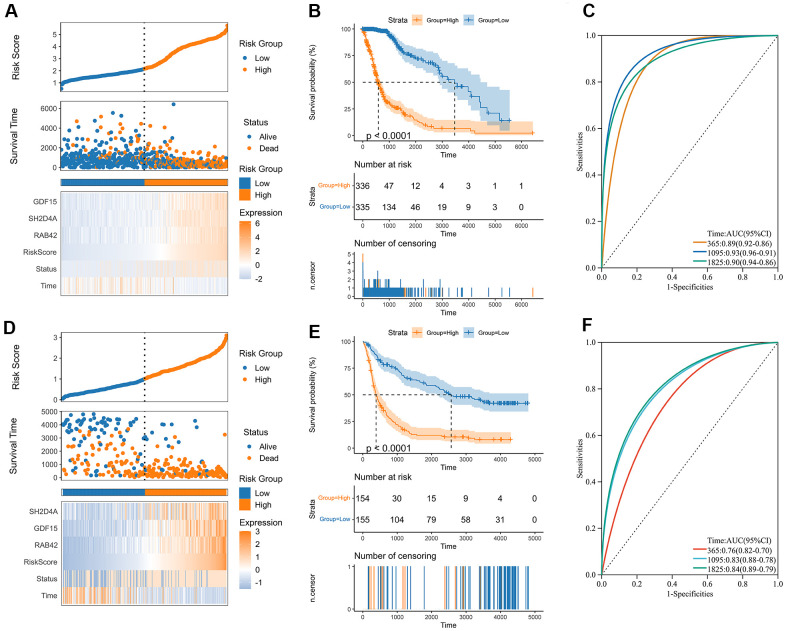
**Construction of the risk score signature.** (**A**) Distribution of the risk score, survival status, and expression profile of the prognostic genes in the TCGA cohort. (**B**) Kaplan-Meier curves displaying prognostic differences between high and low-risk groups in the TCGA cohort. (**C**) The ROC curves describing the sensitivity and specificity of the risk score in predicting OS at 1-, 3- and 5-year time points in the TCGA cohort. (**D**) Distribution of the risk score, survival status, and expression profile of the prognostic genes in the CGGA cohort. (**E**) Kaplan-Meier curves displaying prognostic differences between high and low-risk groups in the CGGA cohort. (**F**) The ROC curves describing the sensitivity and specificity of the risk score in predicting OS at 1-, 3- and 5-year time points in the CGGA cohort.

**Figure 6:**
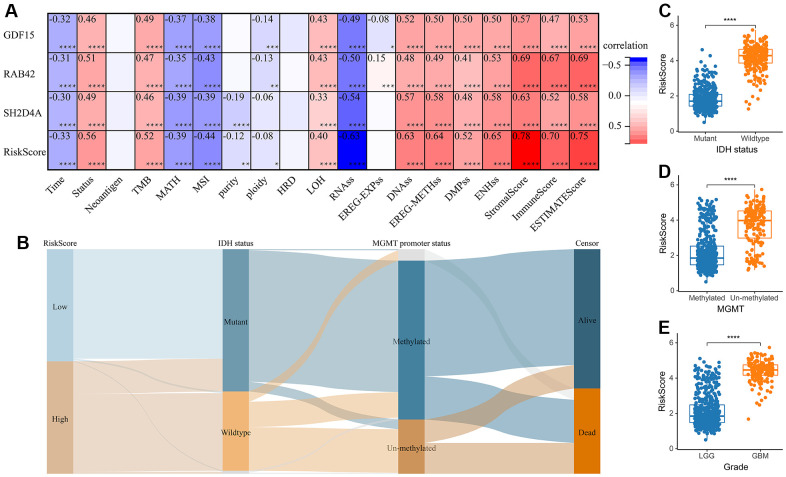
**The relationship between risk score and clinical phenotype.** (**A**) Correlations between 3 risk hub genes and tumor stemness, genomic heterogeneity, and immune microenvironment. (**B**) Sankey Diagram displayed the distribution of the survival status, IDH status, WHO grade, risk score and MGMT promoter status. (**C**) Analysis of the risk scores in different IDH status, (**D**) MGMT promoter status, and (**E**) WHO grades, respectively. *, P < 0.05; **, P < 0.01; ***, P < 0.001; ****, P < 0.0001.

A risk model was then constructed in the TCGA dataset using the risk hub genes RAB42, SH2D4A, and GDF15. Kaplan-Meier curve showed that high-risk scores were associated with poor prognosis. The risk score had high sensitivity and specificity in predicting 1-, 3-, and 5-year survival rates in glioma patients (Figure 5A–5C). The results of the risk model were also confirmed in the CGGA dataset (Figure 5D–5F). In addition, we also validated the risk model in GBM and LGG, respectively (Supplementary Figure 1). The results of the correlation analysis showed that risk scores and risk hub genes RAB42, SH2D4A and GDF15 were highly correlated with the clinical traits such as TMB, MSI, RNAss, DNAss, StromalScore, ImmuneScore and ESTIMATEScore (Figure 6A). To better understand the relationship between the risk signature and clinical characteristics, we analyzed the distribution of survival status, WHO classification, risk scores, MGMT, and IDH status of glioma patients. We identified significant differences in clinical characteristics between patients with low-risk scores and those with high-risk scores. Patients in the low-risk score group exhibited an IDH-Mutant, Methylated phenotype and had a better prognosis, and GBM had a significantly higher risk score than the corresponding LGG subtype (Figure 6B–6E).

### The risk score is an independent risk factor for glioma prognosis

**Figure 7:**
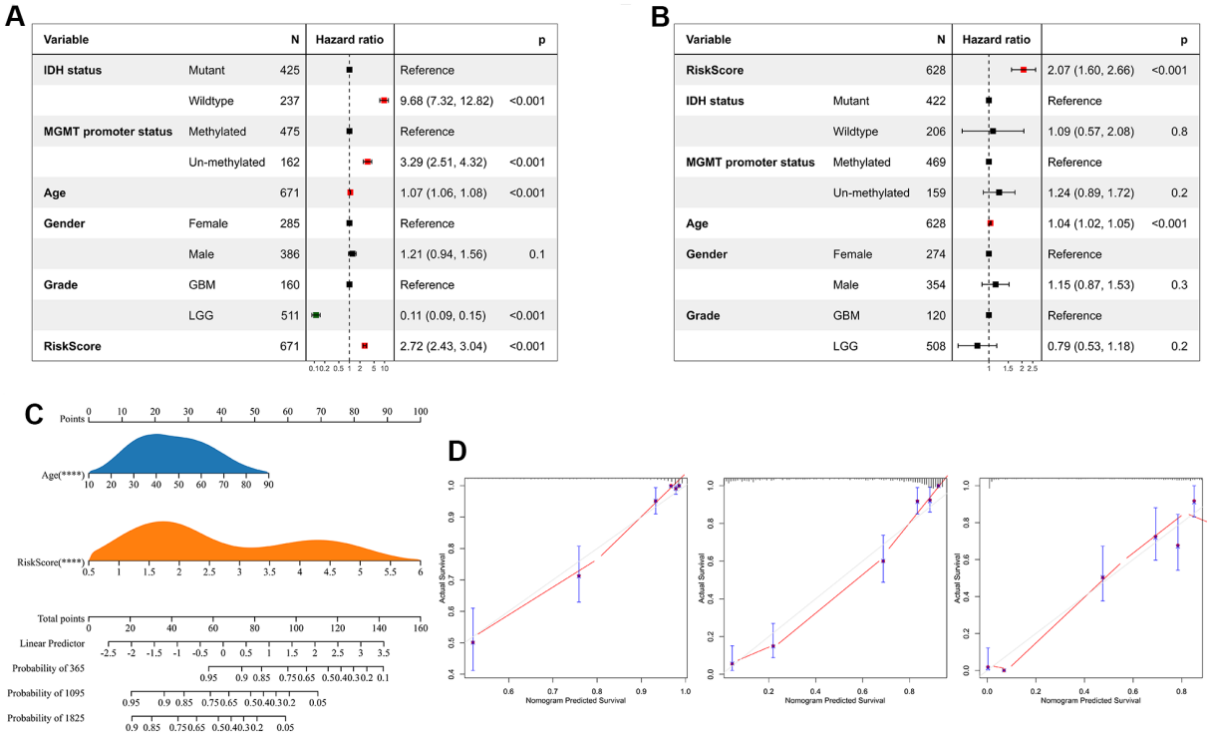
**Risk score is an independent prognostic factor for glioma.** (**A**) Univariate Cox regression analyses showed that clinical features such as the risk score, age, MGMT promoter status, WHO grade, and IDH status were significantly correlated with prognosis. (**B**) Multivariate Cox analysis showed the risk score remained associated with the prognosis. (**C**) The Nomogram was constructed to predict prognosis in patients at 1-, 3-, and 5 years in the TCGA dataset. (**D**) The calibration curve of the nomogram.

We performed univariate Cox regression analysis to investigate the independent prognostic factors for glioma. And the analysis showed that risk score, age, MGMT promoter status, WHO classification, and IDH status were significantly associated with prognosis (Figure 7A). Multivariate Cox regression analysis showed that risk score and age were significantly associated with prognosis, suggesting that risk score is an independent prognostic factor for glioma (Figure 7B). Following that, we developed a nomogram survival prediction model for glioma patients based on independent prognostic parameters, and plotted calibration curves. The results showed good agreement between the predicted outcome and the 1-, 3- and 5-year overall survival of patients (Figure 7C, 7D).

### Risk signature is highly associated with tumor immune cells

**Figure 8:**
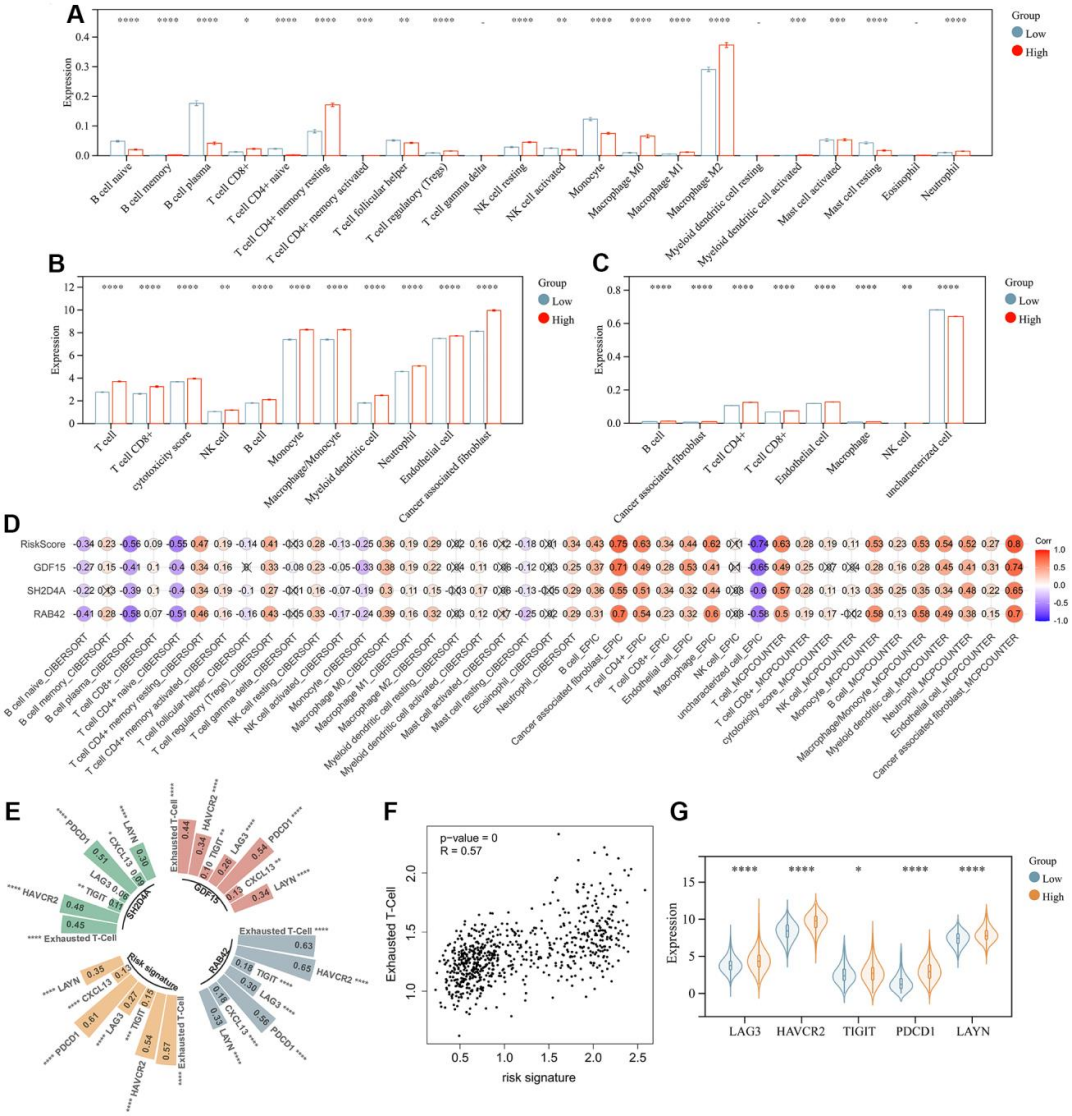
**Risk signature is associated with infiltrating immune cells.** (**A**) The level of immune cell infiltration between different risk subgroups evaluated by CIBERSORT, (**B**) MCPCOUNTER and (**C**) EPIC algorithm, respectively. (**D**) Correlation of infiltrating immune cells with risk scores and 3 risk hub genes. (**E**, **F**) Correlation of exhausted T cell signature with risk scores and 3 risk hub genes. (**G**) Violin plot showing the expression levels of the exhausted markers between low- and high-risk groups in the TCGA dataset. *, P < 0.05; **, P < 0.01; ***, P < 0.001; ****, P < 0.0001.

The above analysis mentioned that DEGs were enriched in immunoregulatory process, and risk signature was highly correlated with the immune microenvironment, so we further analyzed the relationship between risk signature and infiltrating tumor immune cells. Infiltrating analysis showed elevated infiltration of T cell regulatory (Tregs), tumor associated macrophages, tumor associated fibroblasts, neutrophils and endothelial cells in patients in the high-risk score group compared to patients in the low-risk score group (Figure 8A–8C). Moreover, the risk signature and risk hub genes RAB42, SH2D4A, and GDF15 were highly positively correlated with the increase of these immunosuppressive tumor infiltrating cells (Figure 8D). Although CD4+ and CD8+ T cells increased in high-risk group, they might be exhausted T cells, characterized by progressive loss of T cell function and ultimately loss of cascade response. Therefore, we analyzed the relationship between risk signature and T cell exhaustion. The results showed that the risk signature was positively correlated with exhausted T cell signature (Figure 8E, 8F), and the expression of exhausted markers HAVCR2, TIGIT, LAG3, PDCD1, and LAYN was up-regulated in high-risk group (Figure 8G).

### Risk signature is associated with immunotherapy outcome

**Figure 9:**
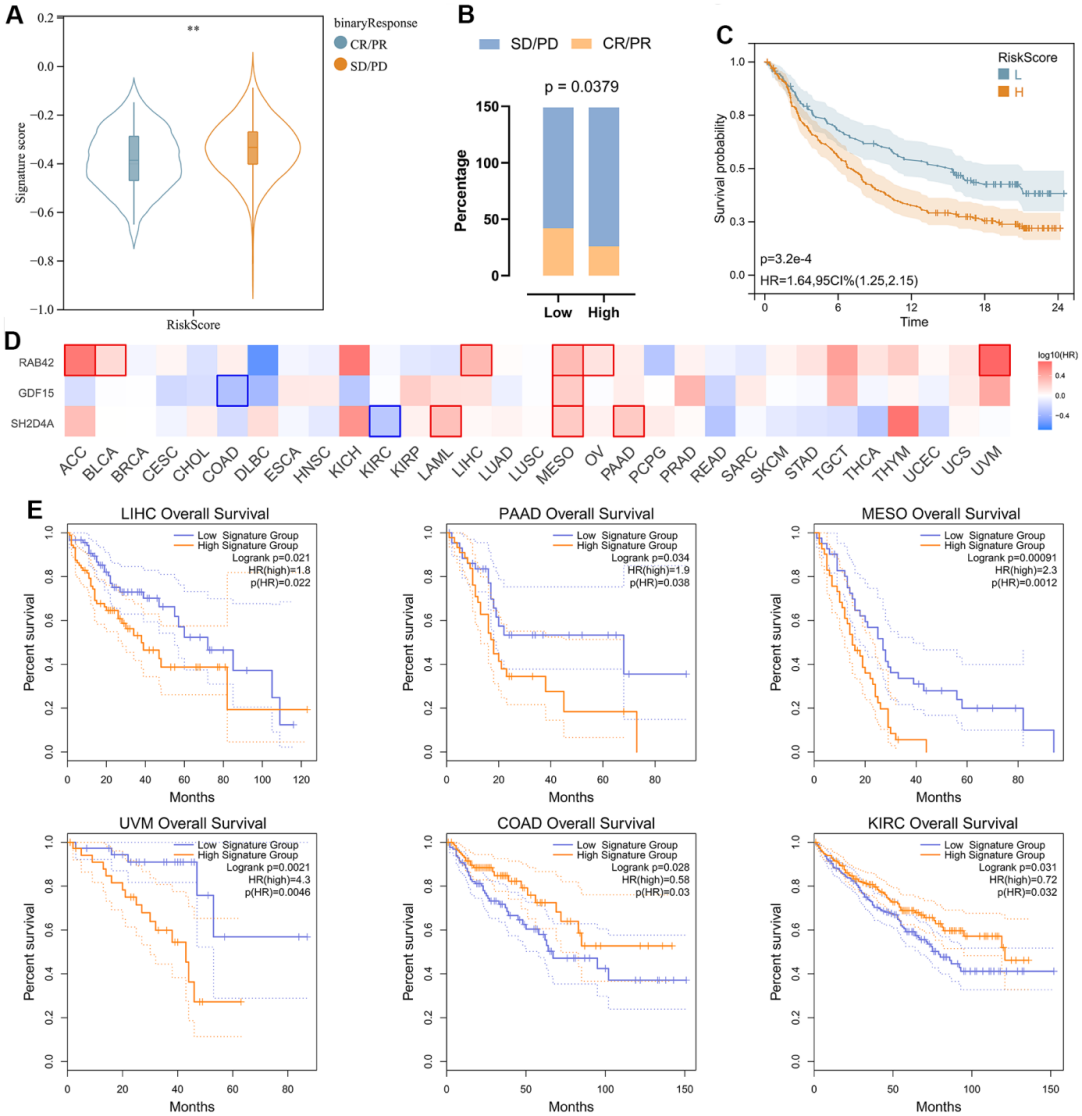
**Analysis of risk signature in immunotherapy cohort and pan cancer.** (**A**) Violin plot depicting the risk scores between SD/PD and CR/PR groups in the IMVIgor210 cohort. (**B**) The response rate of the high-risk score group and low-risk score group to immune therapy in the IMVIgor210 cohort. (**C**) Survival analysis of patients with low-risk scores and high-risk scores in the IMVIgor210 cohort. (**D**) Survival analysis of 3 risk hub genes in pan cancer. (**E**) Kaplan-Meier survival curve of patients in high- and low-risk score groups in pan cancer. **, P < 0.01.

In order to analyze the predictive effect of risk signature on the efficacy of immune checkpoint inhibitors (ICI) therapy, we downloaded gene expression profiles and clinical data of the IMvigor210 cohort. All samples were divided into high and low-risk groups according to the risk signature. Patients with treatment response [complete remission (CR) or partial remission (PR)] had significantly lower risk scores than those without response [stable (SD) or progressive (PD)] (Figure 9A). By evaluating the distribution of CR/PR and SD/PD in the high-risk and low-risk groups, we found that the low-risk group responded better to ICI treatment than the high-risk group (Figure 9B), and had a significantly better prognosis than the high-risk group (Figure 9C). We then analyzed the prognostic value of risk signature and risk hub genes in pan-cancer (Figure 9D, 9E), and the results showed that the low-risk group had a better prognosis in LIHC\PAAD\MESO\UVM (Figure 9E).

### SH2D4A affects the migration and proliferation of glioma cells

**Figure 10:**
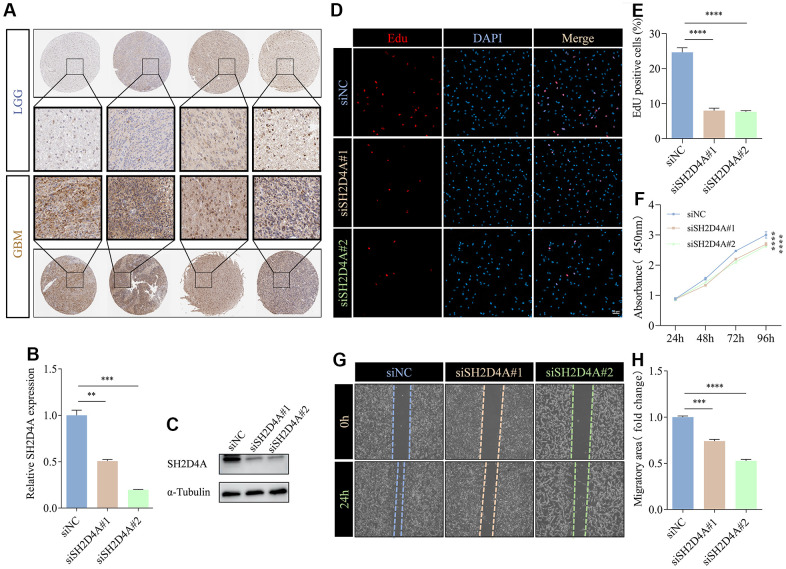
**SH2D4A affects proliferation and migration of glioma cells.** (**A**) Immunohistochemical staining of SH2D4A in glioma (data from HPA). (**B**, **C**) qRT-PCR and western blot analysis of SH2D4A knockdown efficiency in U87 MG cells. (**D**) Analysis of proliferation of control and SH2D4A-deficient U87 MG cells by CCK8 assay. (**E**, **F**) Representative images and statistical analysis of EdU assay in control and SH2D4A-deficient U87 MG cells. (**G**, **H**) Representative images and statistical analysis of cell migration assay in control and SH2D4A-deficient U87 MG cells at the indicated times. **, P < 0.01; ***, P < 0.001; ****, P < 0.0001.

To validate the expression of the risk hub genes in glioma, we analyzed the immunohistochemistry pathological specimen data. The results showed that the expression level of SH2D4A was increased in GBM relative to LGG (Figure 10A). However, the function of SH2D4A in glioma has not been reported in the literature so far. To investigate the effect of SH2D4A on glioma cells, we knocked down SH2D4A in U87 MG cells by transfecting specific siRNAs (Figure 10B, 10C). CCK-8 and Edu assays suggested that SH2D4A significantly affected the proliferation of U87 MG cells (Figure 10D–10F). Cell migration assays confirmed that knockdown of SH2D4A significantly inhibited the migration of U87 MG cells (Figure 10G–10H).

## DISCUSSION

Primary central nervous system tumors arise from heterogeneous cells within the central nervous system (CNS) [40]. Although traditional treatments for glioma, such as surgical resection, temozolomide (TMZ), and radiation therapy, have been used, they offer limited progress in combating the tumor’s progression. Immunotherapy is a recent breakthrough in cancer treatment and is currently at the forefront of cancer therapy [41]. However, it still requires further improvement for gliomas [16, 17]. Previous studies have shown that there is significant genetic, epigenetic, and microenvironmental heterogeneity in each glioma. Intratumoral heterogeneity has a significant influence on tumor recurrence in gliomas, and this presents various challenges in developing targeted therapies [42]. Thus, improvement of patient prognosis requires a better understanding of the genomic heterogeneity, stemness, and immune microenvironment of gliomas.

To address this issue, we conducted a consensus clustering analysis of gliomas’ genomic heterogeneity, stemness, and microenvironment. The results showed that all samples can be classified into subgroups, and the prognostic differences between them were significant (Figure 1). Therefore, identification of driver hub genes affecting these three aspects may help to address treatment failure. Interestingly, genes differentially expressed in different groups in all three consensus clustering analyses were enriched in immunomodulatory interactions (Figure 2E), suggesting that genomic heterogeneity, stemness, and the tumor microenvironment may all contribute to reprogramming the immune status of gliomas, which may have implications for therapy.

Through WGCNA, LASSO and multivariate Cox regression analyses, we identified RAB42, SH2D4A and GDF15 as high-risk hub genes for glioma, and we employed these genes to create a risk model that showed high sensitivity and specificity in prognosticating patients’ 1-, 3-, and 5-year survival (Figure 5). Undoubtedly, the risk model showed an extremely strong correlation with genomic heterogeneity, stemness, and immune microenvironment scores (Figure 6). In addition, we found that the risk signature was closely associated with T cell exhaustion (Figure 8E–8G), which could theoretically be partially reversed by inhibiting the PD-1 pathway. However, we also found that immunosuppressive cells, such as tumor-associated macrophages, tumor-associated fibroblasts, and T cell regulatory (Tregs), were elevated in the high-risk group (Figure 8A–8C). The presence of these immunosuppressive cells may be an important contributor to T-cell exhaustion and to the low response rate to immune checkpoint inhibitor therapy. And these results further corroborate the influence of genomic heterogeneity, stemness and tumor microenvironment on the immune status of glioma (Figures 2, 8).

Studies have shown that RAB42 can promote the proliferation, migration and invasion of glioma cells [43, 44]. And in addition to these functions, GDF15 can regulate immune infiltration of glioma and promote stem cell-like phenotype [45, 46]. However, the role of SH2D4A in glioma remains unclear. SH2D4A is localized to human chromosome 8p21.3 and encodes SH(2)A. Earlier studies indicate that chromosome regions harboring SH2D4A are frequently deleted in various cancer types, and the deletion or downregulation of this gene is related to poor survival and hepatocarcinogenesis [26, 47]. In addition, SH2D4A was found to promote the oncogenic progression of HCT15 and LoVo CRC cells [16]. Given the conflicting roles of SH2D4A in different tumor types, we validated its effects on glioma cells. Our results suggest that SH2D4A contributes to the migration and proliferation of glioma cells (Figure 10). However, the effects and mechanisms of SH2D4A on glioma stemness and microenvironment needs to be further investigated.

## CONCLUSIONS

In this study, we utilized consensus clustering analysis of genome heterogeneity, stemness, and tumor microenvironment to identify hub genes associated with risk in glioma. We then constructed a risk model capable of accurately predicting glioma prognosis and treatment response to immunotherapy. Our findings could pave the way for new strategies to treat glioma.

## Supplementary Material

Supplementary Figure 1

Supplementary Table 1

Supplementary Table 2

Supplementary Tables 3 and 4

## References

[1] Rouse C, Gittleman H, Ostrom QT, Kruchko C, Barnholtz-Sloan JS. Years of potential life lost for brain and CNS tumors relative to other cancers in adults in the United States, 2010. Neuro Oncol. 2016; 18:70–7. 10.1093/neuonc/nov249 26459813 PMC4677421

[2] Zhang N, Zhang L, Qiu B, Meng L, Wang X, Hou BL. Correlation of volume transfer coefficient Ktrans with histopathologic grades of gliomas. J Magn Reson Imaging. 2012; 36:355–63. 10.1002/jmri.23675 22581762 PMC3399966

[3] Ostrom QT, Bauchet L, Davis FG, Deltour I, Fisher JL, Langer CE, Pekmezci M, Schwartzbaum JA, Turner MC, Walsh KM, Wrensch MR, Barnholtz-Sloan JS. The epidemiology of glioma in adults: a “state of the science” review. Neuro Oncol. 2014; 16:896–913. 10.1093/neuonc/nou087 24842956 PMC4057143

[4] Ostrom QT, Gittleman H, Truitt G, Boscia A, Kruchko C, Barnholtz-Sloan JS. CBTRUS Statistical Report: Primary Brain and Other Central Nervous System Tumors Diagnosed in the United States in 2011-2015. Neuro Oncol. 2018; 20:iv1–v86. 10.1093/neuonc/noy131 30445539 PMC6129949

[5] van den Bent MJ. Interobserver variation of the histopathological diagnosis in clinical trials on glioma: a clinician’s perspective. Acta Neuropathol. 2010; 120:297–304. 10.1007/s00401-010-0725-7 20644945 PMC2910894

[6] Suzuki H, Aoki K, Chiba K, Sato Y, Shiozawa Y, Shiraishi Y, Shimamura T, Niida A, Motomura K, Ohka F, Yamamoto T, Tanahashi K, Ranjit M, et al. Mutational landscape and clonal architecture in grade II and III gliomas. Nat Genet. 2015; 47:458–68. 10.1038/ng.3273 25848751

[7] Baumert BG, Hegi ME, van den Bent MJ, von Deimling A, Gorlia T, Hoang-Xuan K, Brandes AA, Kantor G, Taphoorn MJ, Hassel MB, Hartmann C, Ryan G, Capper D, et al. Temozolomide chemotherapy versus radiotherapy in high-risk low-grade glioma (EORTC 22033-26033): a randomised, open-label, phase 3 intergroup study. Lancet Oncol. 2016; 17:1521–32. 10.1016/S1470-2045(16)30313-8 27686946 PMC5124485

[8] Ostrom QT, Cioffi G, Gittleman H, Patil N, Waite K, Kruchko C, Barnholtz-Sloan JS. CBTRUS Statistical Report: Primary Brain and Other Central Nervous System Tumors Diagnosed in the United States in 2012-2016. Neuro Oncol. 2019 (Suppl 5); 21:v1–100. 10.1093/neuonc/noz150 31675094 PMC6823730

[9] Ostrom QT, Gittleman H, Xu J, Kromer C, Wolinsky Y, Kruchko C, Barnholtz-Sloan JS. CBTRUS Statistical Report: Primary Brain and Other Central Nervous System Tumors Diagnosed in the United States in 2009-2013. Neuro Oncol. 2016; 18:v1–75. 10.1093/neuonc/now207 28475809 PMC8483569

[10] Franceschi E, Tosoni A, Bartolini S, Minichillo S, Mura A, Asioli S, Bartolini D, Gardiman M, Gessi M, Ghimenton C, Giangaspero F, Lanza G, Marucci G, et al. Histopathological grading affects survival in patients with IDH-mutant grade II and grade III diffuse gliomas. Eur J Cancer. 2020; 137:10–7. 10.1016/j.ejca.2020.06.018 32721633

[11] Stupp R, Mason WP, van den Bent MJ, Weller M, Fisher B, Taphoorn MJ, Belanger K, Brandes AA, Marosi C, Bogdahn U, Curschmann J, Janzer RC, Ludwin SK, et al, European Organisation for Research and Treatment of Cancer Brain Tumor and Radiotherapy Groups, and National Cancer Institute of Canada Clinical Trials Group. Radiotherapy plus concomitant and adjuvant temozolomide for glioblastoma. N Engl J Med. 2005; 352:987–96. 10.1056/NEJMoa043330 15758009

[12] Molenaar RJ, Radivoyevitch T, Maciejewski JP, van Noorden CJ, Bleeker FE. The driver and passenger effects of isocitrate dehydrogenase 1 and 2 mutations in oncogenesis and survival prolongation. Biochim Biophys Acta. 2014; 1846:326–41. 10.1016/j.bbcan.2014.05.004 24880135

[13] Wang Z, Bao Z, Yan W, You G, Wang Y, Li X, Zhang W. Isocitrate dehydrogenase 1 (IDH1) mutation-specific microRNA signature predicts favorable prognosis in glioblastoma patients with IDH1 wild type. J Exp Clin Cancer Res. 2013; 32:59. 10.1186/1756-9966-32-59 23988086 PMC3847806

[14] Ballabh P, Braun A, Nedergaard M. The blood-brain barrier: an overview: structure, regulation, and clinical implications. Neurobiol Dis. 2004; 16:1–13. 10.1016/j.nbd.2003.12.016 15207256

[15] Oberoi RK, Parrish KE, Sio TT, Mittapalli RK, Elmquist WF, Sarkaria JN. Strategies to improve delivery of anticancer drugs across the blood-brain barrier to treat glioblastoma. Neuro Oncol. 2016; 18:27–36. 10.1093/neuonc/nov164 26359209 PMC4677418

[16] Xu S, Tang L, Li X, Fan F, Liu Z. Immunotherapy for glioma: Current management and future application. Cancer Lett. 2020; 476:1–12. 10.1016/j.canlet.2020.02.002 32044356

[17] Reardon DA, Brandes AA, Omuro A, Mulholland P, Lim M, Wick A, Baehring J, Ahluwalia MS, Roth P, Bähr O, Phuphanich S, Sepulveda JM, De Souza P, et al. Effect of Nivolumab vs Bevacizumab in Patients With Recurrent Glioblastoma: The CheckMate 143 Phase 3 Randomized Clinical Trial. JAMA Oncol. 2020; 6:1003–10. 10.1001/jamaoncol.2020.1024 32437507 PMC7243167

[18] Johnson KC, Anderson KJ, Courtois ET, Gujar AD, Barthel FP, Varn FS, Luo D, Seignon M, Yi E, Kim H, Estecio MR, Zhao D, Tang M, et al. Single-cell multimodal glioma analyses identify epigenetic regulators of cellular plasticity and environmental stress response. Nat Genet. 2021; 53:1456–68. 10.1038/s41588-021-00926-8 34594038 PMC8570135

[19] Weller M, van den Bent M, Tonn JC, Stupp R, Preusser M, Cohen-Jonathan-Moyal E, Henriksson R, Le Rhun E, Balana C, Chinot O, Bendszus M, Reijneveld JC, Dhermain F, et al, and European Association for Neuro-Oncology (EANO) Task Force on Gliomas. European Association for Neuro-Oncology (EANO) guideline on the diagnosis and treatment of adult astrocytic and oligodendroglial gliomas. Lancet Oncol. 2017; 18:e315–29. 10.1016/S1470-2045(17)30194-8 28483413

[20] Louis DN, Perry A, Reifenberger G, von Deimling A, Figarella-Branger D, Cavenee WK, Ohgaki H, Wiestler OD, Kleihues P, Ellison DW. The 2016 World Health Organization Classification of Tumors of the Central Nervous System: a summary. Acta Neuropathol. 2016; 131:803–20. 10.1007/s00401-016-1545-1 27157931

[21] Barthel L, Hadamitzky M, Dammann P, Schedlowski M, Sure U, Thakur BK, Hetze S. Glioma: molecular signature and crossroads with tumor microenvironment. Cancer Metastasis Rev. 2022; 41:53–75. 10.1007/s10555-021-09997-9 34687436 PMC8924130

[22] Ribeiro Franco PI, Rodrigues AP, de Menezes LB, Pacheco Miguel M. Tumor microenvironment components: Allies of cancer progression. Pathol Res Pract. 2020; 216:152729. 10.1016/j.prp.2019.152729 31735322

[23] Poon CC, Sarkar S, Yong VW, Kelly JJ. Glioblastoma-associated microglia and macrophages: targets for therapies to improve prognosis. Brain. 2017; 140:1548–60. 10.1093/brain/aww355 28334886

[24] Arneth B. Tumor Microenvironment. Medicina (Kaunas). 2019; 56:15. 10.3390/medicina56010015 31906017 PMC7023392

[25] Cole AP, Hoffmeyer E, Chetty SL, Cruz-Cruz J, Hamrick F, Youssef O, Cheshier S, Mitra SS. Microglia in the Brain Tumor Microenvironment. Adv Exp Med Biol. 2020; 1273:197–208. 10.1007/978-3-030-49270-0_11 33119883

[26] Quagliata L, Andreozzi M, Kovac M, Tornillo L, Makowska Z, Moretti F, Heim MH, Heinimann K, Piscuoglio S, Terracciano LM. SH2D4A is frequently downregulated in hepatocellular carcinoma and cirrhotic nodules. Eur J Cancer. 2014; 50:731–8. 10.1016/j.ejca.2013.11.018 24315626

[27] Kim JC, Kim JH, Ha YJ, Kim CW, Tak KH, Yoon YS, Kwon YH, Roh SA, Cho DH, Kim SK, Kim SY, Kim YS. Analysis of genomic pathogenesis according to the revised Bethesda guidelines and additional criteria. J Cancer Res Clin Oncol. 2021; 147:117–28. 10.1007/s00432-020-03391-8 32960359 PMC11802017

[28] Cerami E, Gao J, Dogrusoz U, Gross BE, Sumer SO, Aksoy BA, Jacobsen A, Byrne CJ, Heuer ML, Larsson E, Antipin Y, Reva B, Goldberg AP, et al. The cBio cancer genomics portal: an open platform for exploring multidimensional cancer genomics data. Cancer Discov. 2012; 2:401–4. 10.1158/2159-8290.CD-12-0095 22588877 PMC3956037

[29] Zhao Z, Meng F, Wang W, Wang Z, Zhang C, Jiang T. Comprehensive RNA-seq transcriptomic profiling in the malignant progression of gliomas. Sci Data. 2017; 4:170024. 10.1038/sdata.2017.24 28291232 PMC5349247

[30] Mariathasan S, Turley SJ, Nickles D, Castiglioni A, Yuen K, Wang Y, Kadel EE II, Koeppen H, Astarita JL, Cubas R, Jhunjhunwala S, Banchereau R, Yang Y, et al. TGFβ attenuates tumour response to PD-L1 blockade by contributing to exclusion of T cells. Nature. 2018; 554:544–8. 10.1038/nature25501 29443960 PMC6028240

[31] Beroukhim R, Mermel CH, Porter D, Wei G, Raychaudhuri S, Donovan J, Barretina J, Boehm JS, Dobson J, Urashima M, Mc Henry KT, Pinchback RM, Ligon AH, et al. The landscape of somatic copy-number alteration across human cancers. Nature. 2010; 463:899–905. 10.1038/nature08822 20164920 PMC2826709

[32] Bonneville R, Krook MA, Kautto EA, Miya J, Wing MR, Chen HZ, Reeser JW, Yu L, Roychowdhury S. Landscape of Microsatellite Instability Across 39 Cancer Types. JCO Precis Oncol. 2017; 2017. 10.1200/PO.17.00073 29850653 PMC5972025

[33] Thorsson V, Gibbs DL, Brown SD, Wolf D, Bortone DS, Ou Yang TH, Porta-Pardo E, Gao GF, Plaisier CL, Eddy JA, Ziv E, Culhane AC, Paull EO, et al, and Cancer Genome Atlas Research Network. The Immune Landscape of Cancer. Immunity. 2018; 48:812–30.e14. 10.1016/j.immuni.2018.03.023 29628290 PMC5982584

[34] Malta TM, Sokolov A, Gentles AJ, Burzykowski T, Poisson L, Weinstein JN, Kamińska B, Huelsken J, Omberg L, Gevaert O, Colaprico A, Czerwińska P, Mazurek S, et al, and Cancer Genome Atlas Research Network. Machine Learning Identifies Stemness Features Associated with Oncogenic Dedifferentiation. Cell. 2018; 173:338–54.e15. 10.1016/j.cell.2018.03.034 29625051 PMC5902191

[35] Yoshihara K, Shahmoradgoli M, Martínez E, Vegesna R, Kim H, Torres-Garcia W, Treviño V, Shen H, Laird PW, Levine DA, Carter SL, Getz G, Stemke-Hale K, et al. Inferring tumour purity and stromal and immune cell admixture from expression data. Nat Commun. 2013; 4:2612. 10.1038/ncomms3612 24113773 PMC3826632

[36] Li T, Fu J, Zeng Z, Cohen D, Li J, Chen Q, Li B, Liu XS. TIMER2.0 for analysis of tumor-infiltrating immune cells. Nucleic Acids Res. 2020; 48:W509–14. 10.1093/nar/gkaa407 32442275 PMC7319575

[37] Wilkerson MD, Hayes DN. ConsensusClusterPlus: a class discovery tool with confidence assessments and item tracking. Bioinformatics. 2010; 26:1572–3. 10.1093/bioinformatics/btq170 20427518 PMC2881355

[38] Shen C, Yu J, Zhang X, Liu CC, Guo YS, Zhu JW, Zhang K, Yu Y, Gao TT, Yang SM, Li H, Zheng B, Huang XY. Strawberry Notch 1 (SBNO1) promotes proliferation of spermatogonial stem cells via the noncanonical Wnt pathway in mice. Asian J Androl. 2019; 21:345–50. 10.4103/aja.aja_65_18 30198493 PMC6628735

[39] Zhou Y, Zhou B, Pache L, Chang M, Khodabakhshi AH, Tanaseichuk O, Benner C, Chanda SK. Metascape provides a biologist-oriented resource for the analysis of systems-level datasets. Nat Commun. 2019; 10:1523. 10.1038/s41467-019-09234-6 30944313 PMC6447622

[40] Lapointe S, Perry A, Butowski NA. Primary brain tumours in adults. Lancet. 2018; 392:432–46. 10.1016/S0140-6736(18)30990-5 30060998

[41] Nabors LB, Portnow J, Ammirati M, Baehring J, Brem H, Butowski N, Fenstermaker RA, Forsyth P, Hattangadi-Gluth J, Holdhoff M, Howard S, Junck L, Kaley T, et al. NCCN Guidelines Insights: Central Nervous System Cancers, Version 1.2017. J Natl Compr Canc Netw. 2017; 15:1331–45. 10.6004/jnccn.2017.0166 29118226

[42] Nicholson JG, Fine HA. Diffuse Glioma Heterogeneity and Its Therapeutic Implications. Cancer Discov. 2021; 11:575–90. 10.1158/2159-8290.CD-20-1474 33558264

[43] Sun L, Yan T, Yang B. The Progression Related Gene *RAB42* Affects the Prognosis of Glioblastoma Patients. Brain Sci. 2022; 12:767. 10.3390/brainsci12060767 35741652 PMC9220890

[44] Liu B, Su Q, Xiao B, Zheng G, Zhang L, Yin J, Wang L, Che F, Heng X. RAB42 Promotes Glioma Pathogenesis via the VEGF Signaling Pathway. Front Oncol. 2021; 11:657029. 10.3389/fonc.2021.657029 Erratum in: Front Oncol. 2022; 12:1034167. 10.3389/fonc.2021.657029 34912698 PMC8666624

[45] Zhu S, Yang N, Guan Y, Wang X, Zang G, Lv X, Deng S, Wang W, Li T, Chen J. GDF15 promotes glioma stem cell-like phenotype via regulation of ERK1/2-c-Fos-LIF signaling. Cell Death Discov. 2021; 7:3. 10.1038/s41420-020-00395-8 33431816 PMC7801449

[46] Hasanpour Segherlou Z, Nouri-Vaskeh M, Noroozi Guilandehi S, Baghbanzadeh A, Zand R, Baradaran B, Zarei M. GDF-15: Diagnostic, prognostic, and therapeutic significance in glioblastoma multiforme. J Cell Physiol. 2021; 236:5564–81. 10.1002/jcp.30289 33580506

[47] Ploeger C, Huth T, Sugiyanto RN, Pusch S, Goeppert B, Singer S, Tabti R, Hausser I, Schirmacher P, Désaubry L, Roessler S. Prohibitin, STAT3 and SH2D4A physically and functionally interact in tumor cell mitochondria. Cell Death Dis. 2020; 11:1023. 10.1038/s41419-020-03220-3 33257655 PMC7705682

