# Supplementary material for: Integrated clustering signature of genomic heterogeneity, stemness and tumor microenvironment predicts glioma prognosis and immunotherapy response
Source: Aging (Albany NY). 2023 Sep 11;15(17):9086–104. doi: 10.18632/aging.205018 (PMC10522363; doi:10.18632/aging.205018)
Supplement: Supplementary Figure 1 [file aging-15-205018-s001.pdf]

SUPPLEMENTARY FIGURE

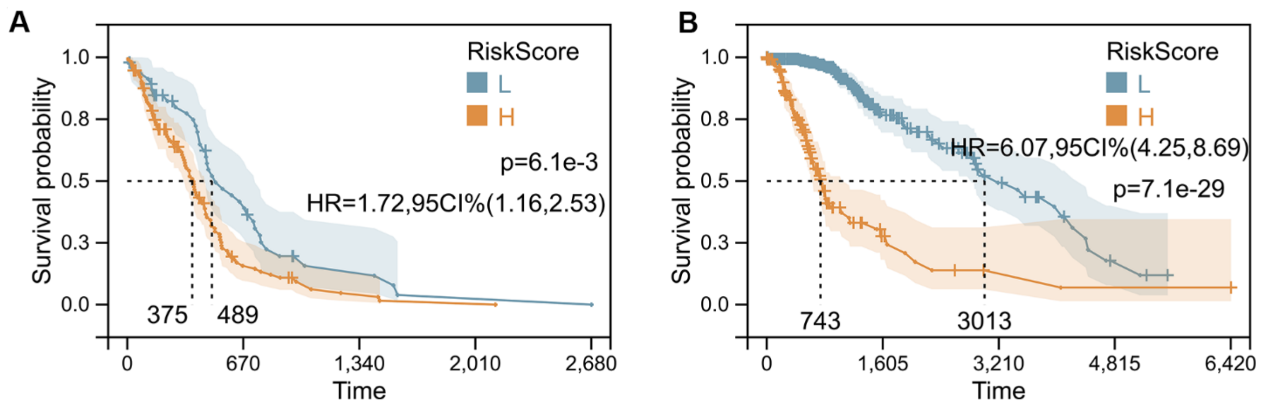

Supplementary Figure 1. Validation of risk model in GBM and LGG. (**A**) Kaplan-Meier curves displaying prognostic differences between high- and low-risk groups in GBM and (**B**) LGG cohorts, respectively.
