# Supplementary material for: Integrated clustering signature of genomic heterogeneity, stemness and tumor microenvironment predicts glioma prognosis and immunotherapy response
Source: Aging (Albany NY). 2023 Sep 11;15(17):9086–104. doi: 10.18632/aging.205018 (PMC10522363; doi:10.18632/aging.205018)
Supplement: Supplementary Tables 3 and 4 [file aging-15-205018-s004.pdf]

Supplementary Table 3. siRNA used in this study.

Name	Sequence
siNC-sense	5’-UUCUCCGAACGUGUCACGUTT-3’
siNC-antisense	5’-ACGUGACACGUUCGGAGAATT-3’
siSH2D4A#1-sense	5’-GAUCCGACGAUGGAAAGAATT-3’
siSH2D4A#1-antisense	5’-UUCUUUCCAUCGUCGGAUCTT-3’
siSH2D4A#2-sense	5’-GCGAACACCAUCUAGAUAATT-3’
siSH2D4A#2-antisense	5’-UUAUCUAGAUGGUGUUCGCTT-3’

Supplementary Table 4. qPCR primer list.

Name	Sequence
SH2D4A-F	5’-CTGGAGCAAGGATCGAGGC-3’
SH2D4A -R	5’-CAGCTCTTACAAATCTGCTTCGT-3’
